# Supplementary material for: A Likelihood Approach for Real-Time Calibration of Stochastic Compartmental Epidemic Models
Source: PLoS Comput Biol. 2017 Jan 17;13(1):e1005257. doi: 10.1371/journal.pcbi.1005257 (PMC5240920; doi:10.1371/journal.pcbi.1005257)
Supplement: S1 File — (TAR.GZ) [file pcbi.1005257.s014.tar.gz › HSPH_Online-SI-Revision/IPoi09/n1-severe/n1-severe_BENCH2_table-4.pdf]

1	newly 3 weeks spec	33.	22.8	{1., 45.}	48.9394	0.457615	newly 3 weeks cumul	81.	86.875	{14., 158.}	54.1667	0.293299	newly 5 weeks spec	34.	15.55	{0., 32.}	62.2059	0.832331	newly 5 weeks cumul	158.	120.225	{14., 211.}	43.0854	0.339906	newly 58 weeks cumul	531.	184.525	{14., 351.}	65.2495	0.656474
2	newly 3 weeks spec	55.	17.3704	{0., 43.}	69.3603	1.10776	newly 3 weeks cumul	130.	61.0741	{0., 150.}	60.5413	0.939831	newly 5 weeks spec	53.	10.4074	{0., 29.}	80.3634	1.54936	newly 5 weeks cumul	247.	84.6667	{0., 224.}	66.1419	1.1028	newly 60 weeks cumul	592.	109.481	{0., 324.}	81.5065	1.42899
3	newly 3 weeks spec	32.	34.2	{9., 74.}	54.375	0.262528	newly 3 weeks cumul	74.	107.314	{40., 185.}	64.7104	0.221752	newly 5 weeks spec	43.	28.1429	{3., 58.}	55.1495	0.508987	newly 5 weeks cumul	164.	166.371	{51., 287.}	42.6307	0.213707	newly 59 weeks cumul	553.	270.886	{52., 518.}	53.5986	0.431454
4	newly 3 weeks spec	58.	47.6	{14., 76.}	34.5977	0.25975	newly 3 weeks cumul	144.	109.883	{32., 171.}	37.8819	0.301378	newly 5 weeks spec	59.	66.25	{29., 95.}	34.3785	0.188552	newly 5 weeks cumul	286.	235.083	{75., 346.}	29.4697	0.232272	newly 62 weeks cumul	620.	777.917	{676., 875.}	30.1317	0.170935
5	newly 3 weeks spec	56.	74.0317	{38., 104.}	47.5624	0.186321	newly 3 weeks cumul	140.	198.921	{72., 298.}	60.7256	0.225971	newly 5 weeks spec	40.	77.7937	{53., 98.}	96.7063	0.285672	newly 5 weeks cumul	241.	354.698	{187., 472.}	57.189	0.203887	newly 61 weeks cumul	534.	759.175	{626., 862.}	42.6372	0.151843
6	newly 3 weeks spec	31.	26.375	{5., 50.}	46.1022	0.316708	newly 3 weeks cumul	71.	69.9167	{11., 129.}	49.6479	0.279747	newly 5 weeks spec	40.	30.8958	{5., 53.}	39.7396	0.295419	newly 5 weeks cumul	159.	130.938	{30., 237.}	40.1861	0.269651	newly 58 weeks cumul	620.	597.688	{418., 723.}	13.7937	0.0664185
7	newly 3 weeks spec	37.	60.1833	{40., 78.}	70.4054	0.237711	newly 3 weeks cumul	101.	189.2	{118., 234.}	93.1683	0.293087	newly 5 weeks spec	30.	48.5833	{26., 73.}	72.2778	0.243852	newly 5 weeks cumul	170.	293.833	{196., 368.}	78.9608	0.26175	newly 58 weeks cumul	392.	531.167	{350., 703.}	41.8112	0.147948
8	newly 3 weeks spec	83.	69.7333	{26., 101.}	29.0361	0.18197	newly 3 weeks cumul	181.	175.933	{59., 274.}	37.6243	0.206551	newly 5 weeks spec	97.	74.5667	{48., 92.}	24.433	0.136479	newly 5 weeks cumul	384.	326.2	{169., 446.}	23.316	0.13827	newly 62 weeks cumul	588.	742.55	{611., 864.}	28.4439	0.107161
9	newly 3 weeks spec	38.	49.6275	{15., 72.}	57.4303	0.266683	newly 3 weeks cumul	79.	135.784	{43., 191.}	93.7702	0.324287	newly 5 weeks spec	31.	48.1765	{21., 71.}	70.2087	0.249321	newly 5 weeks cumul	150.	232.843	{101., 327.}	74.9412	0.274162	newly 58 weeks cumul	454.	589.	{390., 741.}	35.7994	0.13032
10	newly 3 weeks spec	67.	64.931	{40., 91.}	22.0793	0.107317	newly 3 weeks cumul	156.	184.138	{95., 241.}	35.3006	0.14822	newly 5 weeks spec	47.	62.6379	{45., 77.}	37.5275	0.135286	newly 5 weeks cumul	273.	312.914	{195., 381.}	25.6347	0.107383	newly 58 weeks cumul	540.	682.966	{554., 813.}	29.6169	0.111283
11	newly 3 weeks spec	25.	42.0833	{19., 62.}	76.3333	0.232971	newly 3 weeks cumul	76.	127.167	{55., 180.}	76.9737	0.246032	newly 5 weeks spec	31.	41.4792	{23., 59.}	44.8253	0.162699	newly 5 weeks cumul	146.	210.146	{120., 295.}	53.2106	0.185441	newly 55 weeks cumul	505.	495.75	{330., 719.}	24.0182	0.116293
12	newly 3 weeks spec	95.	95.7619	{45., 128.}	27.8028	0.143378	newly 3 weeks cumul	232.	236.222	{93., 336.}	33.4975	0.175659	newly 5 weeks spec	59.	93.4127	{68., 115.}	60.9093	0.201915	newly 5 weeks cumul	391.	429.349	{266., 528.}	25.9002	0.119231	newly 63 weeks cumul	569.	805.635	{691., 907.}	41.5879	0.148944
13	newly 3 weeks spec	46.	59.6786	{37., 83.}	42.4689	0.174631	newly 3 weeks cumul	111.	162.929	{89., 219.}	58.7194	0.221133	newly 5 weeks spec	49.	58.0893	{38., 76.}	28.9723	0.133925	newly 5 weeks cumul	221.	279.875	{183., 346.}	37.096	0.158889	newly 58 weeks cumul	585.	628.054	{506., 750.}	16.0958	0.0872023
14	newly 3 weeks spec	59.	67.5614	{24., 104.}	45.6735	0.256715	newly 3 weeks cumul	122.	150.982	{51., 249.}	53.7245	0.263947	newly 5 weeks spec	75.	87.0877	{53., 117.}	30.1053	0.165608	newly 5 weeks cumul	272.	318.491	{151., 457.}	39.7188	0.206131	newly 63 weeks cumul	614.	796.07	{666., 904.}	33.3276	0.165937
15	newly 3 weeks spec	43.	50.9818	{22., 76.}	40.4651	0.165441	newly 3 weeks cumul	132.	170.455	{85., 229.}	41.1983	0.155221	newly 5 weeks spec	36.	38.7273	{17., 64.}	39.798	0.181359	newly 5 weeks cumul	229.	253.745	{132., 347.}	31.5601	0.135935	newly 57 weeks cumul	373.	416.818	{188., 634.}	38.099	0.167391
16	newly 3 weeks spec	37.	75.9667	{25., 114.}	120.541	0.41009	newly 3 weeks cumul	113.	193.05	{57., 297.}	93.5251	0.385719	newly 5 weeks spec	55.	81.35	{58., 102.}	55.9697	0.267218	newly 5 weeks cumul	226.	356.917	{164., 487.}	71.9985	0.333512	newly 63 weeks cumul	577.	740.083	{595., 877.}	35.6008	0.243418
17	newly 3 weeks spec	80.	93.7119	{40., 135.}	37.0551	0.156107	newly 3 weeks cumul	176.	263.949	{120., 356.}	61.1807	0.20879	newly 5 weeks spec	63.	76.1864	{21., 120.}	50.9551	0.278827	newly 5 weeks cumul	335.	428.424	{248., 553.}	38.2595	0.145485	newly 63 weeks cumul	583.	658.729	{344., 866.}	31.055	0.137733
18	newly 3 weeks spec	27.	56.4127	{16., 101.}	130.1	0.45002	newly 3 weeks cumul	67.	131.889	{30., 243.}	122.151	0.430622	newly 5 weeks spec	23.	71.8571	{28., 103.}	226.363	0.57448	newly 5 weeks cumul	126.	265.984	{104., 427.}	127.324	0.441171	newly 63 weeks cumul	593.	750.127	{604., 881.}	34.8055	0.237908
19	newly 3 weeks spec	46.	52.8036	{27., 75.}	31.1724	0.127162	newly 3 weeks cumul	143.	164.75	{96., 222.}	30.0699	0.13239	newly 5 weeks spec	45.	42.9286	{22., 66.}	28.0952	0.136014	newly 5 weeks cumul	247.	257.5	{154., 337.}	22.5564	0.102594	newly 56 weeks cumul	533.	484.911	{348., 703.}	25.4523	0.127889
20	newly 3 weeks spec	63.	61.3731	{39., 80.}	18.171	0.0870338	newly 3 weeks cumul	168.	184.881	{122., 234.}	24.0672	0.106435	newly 5 weeks spec	55.	52.0597	{35., 72.}	18.6974	0.0883414	newly 5 weeks cumul	291.	294.716	{233., 360.}	15.4126	0.0703326	newly 56 weeks cumul	494.	582.224	{416., 756.}	27.189	0.10568
21	newly 3 weeks spec	38.	45.7174	{6., 102.}	80.3776	0.446965	newly 3 weeks cumul	97.	163.196	{30., 291.}	96.7055	0.39142	newly 5 weeks spec	50.	26.	{0., 73.}	70.087	0.954869	newly 5 weeks cumul	199.	226.783	{31., 467.}	65.6762	0.397103	newly 63 weeks cumul	538.	276.304	{31., 661.}	57.0551	0.578553
22	newly 3 weeks spec	77.	58.3833	{38., 75.}	25.	0.1546	newly 3 weeks cumul	210.	164.633	{90., 222.}	23.8254	0.156898	newly 5 weeks spec	61.	56.6	{36., 73.}	19.4536	0.0983165	newly 5 weeks cumul	359.	283.5	{209., 344.}	21.1421	0.125857	newly 57 weeks cumul	537.	646.767	{513., 779.}	22.7126	0.0860998
23	newly 3 weeks spec	30.	24.5	{4., 56.}	60.625	0.397836	newly 3 weeks cumul	92.	99.0938	{35., 186.}	58.322	0.261329	newly 5 weeks spec	37.	15.75	{0., 41.}	65.0338	0.899618	newly 5 weeks cumul	174.	134.281	{38., 266.}	51.9935	0.319939	newly 59 weeks cumul	461.	187.688	{41., 390.}	59.6936	0.533266
24	newly 3 weeks spec	38.	15.0476	{1., 42.}	69.7995	0.836059	newly 3 weeks cumul	121.	59.3333	{8., 139.}	63.0854	0.573364	newly 5 weeks spec	32.	7.35714	{0., 15.}	80.878	1.15669	newly 5 weeks cumul	201.	76.4762	{10., 196.}	66.7851	0.671681	newly 56 weeks cumul	407.	99.2381	{10., 242.}	77.4658	0.876902
25	newly 3 weeks spec	55.	45.1429	{25., 63.}	22.987	0.124722	newly 3 weeks cumul	180.	130.839	{73., 178.}	28.2242	0.164672	newly 5 weeks spec	62.	43.6071	{28., 56.}	29.6659	0.167356	newly 5 weeks cumul	314.	219.946	{141., 280.}	29.9534	0.167762	newly 54 weeks cumul	547.	576.25	{441., 720.}	17.0606	0.073012
26	newly 3 weeks spec	41.	76.	{45., 103.}	88.1636	0.261781	newly 3 weeks cumul	123.	233.735	{152., 293.}	94.6437	0.28598	newly 5 weeks spec	42.	58.75	{35., 82.}	48.7745	0.168992	newly 5 weeks cumul	210.	360.794	{233., 445.}	73.6555	0.23216	newly 61 weeks cumul	500.	601.706	{372., 827.}	30.0471	0.115538
27	newly 3 weeks spec	55.	29.5588	{6., 62.}	57.1658	0.453718	newly 3 weeks cumul	170.	107.059	{52., 188.}	43.3564	0.279268	newly 5 weeks spec	64.	15.5294	{1., 40.}	75.8272	0.980398	newly 5 weeks cumul	324.	144.912	{68., 276.}	56.4724	0.429384	newly 60 weeks cumul	489.	202.529	{71., 425.}	59.8099	0.501038
28	newly 3 weeks spec	52.	70.4861	{20., 113.}	65.9989	0.297107	newly 3 weeks cumul	108.	170.236	{34., 297.}	89.4676	0.353804	newly 5 weeks spec	42.	80.5278	{42., 107.}	99.2725	0.32429	newly 5 weeks cumul	207.	330.083	{122., 485.}	78.7037	0.30642	newly 63 weeks cumul	611.	804.458	{740., 896.}	34.4404	0.175122
29	newly 3 weeks spec	39.	48.5333	{29., 65.}	34.7009	0.128667	newly 3 weeks cumul	112.	159.55	{104., 204.}	45.5804	0.155285	newly 5 weeks spec	44.	38.25	{21., 63.}	33.9773	0.174275	newly 5 weeks cumul	200.	239.233	{154., 312.}	30.3833	0.116808	newly 56 weeks cumul	440.	405.983	{225., 594.}	28.3144	0.136775
30	newly 3 weeks spec	46.	59.8852	{37., 83.}	41.8033	0.154276	newly 3 weeks cumul	145.	197.459	{122., 252.}	44.7258	0.1614	newly 5 weeks spec	43.	44.377	{20., 69.}	37.9718	0.17731	newly 5 weeks cumul	245.	293.213	{185., 378.}	32.9006	0.129687	newly 57 weeks cumul	463.	476.721	{263., 697.}	28.3858	0.130029
31	newly 3 weeks spec	17.	45.8246	{22., 67.}	175.129	0.446129	newly 3 weeks cumul	48.	148.912	{85., 206.}	216.374	0.526542	newly 5 weeks spec	16.	37.0351	{10., 63.}	145.504	0.411274	newly 5 weeks cumul	108.	226.825	{119., 328.}	118.015	0.378595	newly 58 weeks cumul	420.	399.579	{190., 599.}	33.2414	0.218375
32	newly 3 weeks spec	35.	48.1803	{15., 71.}	66.4169	0.308866	newly 3 weeks cumul	65.	121.492	{27., 212.}	112.989	0.373963	newly 5 weeks spec	21.	59.	{20., 83.}	189.539	0.460088	newly 5 weeks cumul	124.	234.787	{63., 347.}	109.81	0.3605	newly 60 weeks cumul	460.	734.197	{562., 846.}	62.7227	0.224458
33	newly 3 weeks spec	48.	85.5909	{31., 120.}	89.2992	0.274855	newly 3 weeks cumul	140.	197.303	{61., 300.}	63.961	0.239116	newly 5 weeks spec	62.	97.4394	{70., 124.}	59.3597	0.196544	newly 5 weeks cumul	272.	389.818	{206., 520.}	53.5762	0.190368	newly 63 weeks cumul	628.	826.5	{672., 900.}	32.5733	0.121116
34	newly 3 weeks spec	16.	27.4468	{5., 51.}	97.3404	0.309904	newly 3 weeks cumul	68.	100.745	{29., 166.}	71.2453	0.251282	newly 5 weeks spec	22.	16.1277	{3., 33.}	57.4468	0.406657	newly 5 weeks cumul	123.	137.617	{39., 239.}	51.0119	0.234405	newly 55 weeks cumul	356.	192.277	{45., 380.}	52.05	0.405962
35	newly 3 weeks spec	73.	73.8596	{19., 116.}	38.236	0.251072	newly 3 weeks cumul	170.	195.982	{43., 323.}	48.4933	0.267714	newly 5 weeks spec	62.	75.2807	{35., 115.}	46.661	0.245331	newly 5 weeks cumul	317.	348.807	{115., 501.}	36.8753	0.212816	newly 63 weeks cumul	588.	686.754	{407., 881.}	31.4566	0.167988
36	newly 3 weeks spec	88.	57.2889	{16., 125.}	54.0404	0.413745	newly 3 weeks cumul	210.	277.444	{125., 436.}	55.3122	0.26725	newly 5 weeks spec	57.	15.6222	{0., 43.}	72.9045	0.955937	newly 5 weeks cumul	377.	323.778	{141., 507.}	35.7501	0.261193	newly 64 weeks cumul	488.	337.467	{144., 547.}	39.2805	0.312421
37	newly 3 weeks spec	41.	47.9057	{30., 67.}	31.8454	0.164566	newly 3 weeks cumul	138.	148.943	{101., 200.}	24.7197	0.137608	newly 5 weeks spec	36.	38.8491	{21., 60.}	35.4822	0.186626	newly 5 weeks cumul	224.	229.321	{159., 306.}	24.5283	0.142868	newly 54 weeks cumul	482.	430.264	{246., 647.}	29.0848	0.186152
38	newly 3 weeks spec	82.	82.6545	{37., 117.}	27.5831	0.165748	newly 3 weeks cumul	203.	223.	{89., 328.}	37.2951	0.204253	newly 5 weeks spec	63.	73.9091	{39., 107.}	39.3074	0.173445	newly 5 weeks cumul	360.	377.836	{237., 498.}	25.5303	0.139625	newly 62 weeks cumul	525.	682.673	{451., 860.}	37.2918	0.137306
39	newly 3 weeks spec	46.	55.8571	{23., 77.}	43.9959	0.204563	newly 3 weeks cumul	116.	156.619	{52., 228.}	58.1418	0.247199	newly 5 weeks spec	36.	55.0794	{32., 74.}	64.903	0.24546	newly 5 weeks cumul	202.	269.365	{125., 362.}	51.0451	0.215589	newly 58 weeks cumul	447.	654.429	{521., 785.}	51.7666	0.196808
40	newly 3 weeks spec	52.	71.6383	{15., 130.}	73.7725	0.291108	newly 3 weeks cumul	151.	284.723	{138., 431.}	93.0393	0.267284	newly 5 weeks spec	41.	27.7234	{1., 73.}	67.7737	0.655339	newly 5 weeks cumul	250.	359.	{140., 542.}	58.5617	0.201881	newly 64 weeks cumul	483.	397.383	{140., 624.}	33.6109	0.194652
41	newly 3 weeks spec	64.	70.5909	{37., 100.}	33.357	0.176064	newly 3 weeks cumul	223.	179.182	{78., 273.}	31.7434	0.212955	newly 5 weeks spec	76.	78.3333	{56., 101.}	20.933	0.101235	newly 5 weeks cumul	393.	335.258	{184., 455.}	21.2006	0.13468	newly 61 weeks cumul	574.	769.03	{653., 871.}	34.3258	0.125869
42	newly 3 weeks spec	30.	62.8889	{9., 102.}	133.037	0.407502	newly 3 weeks cumul	92.	194.667	{70., 288.}	124.155	0.351938	newly 5 weeks spec	39.	46.4	{5., 83.}	68.8889	0.46515	newly 5 weeks cumul	182.	297.022	{79., 425.}	84.5421	0.297191	newly 61 weeks cumul	534.	478.222	{92., 778.}	34.2405	0.229023
43	newly 3 weeks spec	35.	44.5263	{26., 61.}	38.8471	0.146439	newly 3 weeks cumul	108.	134.86	{75., 181.}	39.4574	0.156534	newly 5 weeks spec	36.	41.2632	{27., 57.}	28.2651	0.111456	newly 5 weeks cumul	191.	221.316	{135., 290.}	28.0151	0.115356	newly 54 weeks cumul	401.	553.667	{376., 722.}	41.7815	0.144944
44	newly 3 weeks spec	64.	57.8571	{7., 97.}	42.8253	0.309962	newly 3 weeks cumul	144.	169.429	{54., 302.}	51.9841	0.228682	newly 5 weeks spec	60.	51.6735	{5., 90.}	46.7347	0.449608	newly 5 weeks cumul	278.	277.408	{66., 454.}	40.1336	0.222443	newly 62 weeks cumul	520.	490.408	{91., 807.}	44.2308	0.283962
45	newly 3 weeks spec	36.	70.2909	{34., 94.}	101.919	0.304768	newly 3 weeks cumul	140.	206.6	{121., 287.}	58.4286	0.215521	newly 5 weeks spec	47.	55.6	{32., 78.}	35.8607	0.141013	newly 5 weeks cumul	246.	325.945	{212., 424.}	41.1013	0.156393	newly 59 weeks cumul	529.	565.8	{363., 763.}	25.104	0.108876
46	newly 3 weeks spec	25.	42.4151	{15., 63.}	86.8679	0.340559	newly 3 weeks cumul	61.	127.679	{46., 194.}	126.384	0.412153	newly 5 weeks spec	28.	40.	{19., 59.}	63.3423	0.279158	newly 5 weeks cumul	125.	209.736	{107., 314.}	82.7623	0.326969	newly 58 weeks cumul	517.	487.736	{290., 679.}	24.8422	0.183288
47	newly 3 weeks spec	97.	69.3387	{10., 118.}	39.1919	0.388707	newly 3 weeks cumul	190.	157.161	{24., 295.}	44.9576	0.395411	newly 5 weeks spec	95.	85.2581	{31., 119.}	28.7946	0.291083	newly 5 weeks cumul	400.	323.968	{78., 509.}	32.	0.336255	newly 64 weeks cumul	577.	767.21	{582., 915.}	46.2207	0.316415
48	newly 3 weeks spec	31.	65.6032	{47., 87.}	113.671	0.321033	newly 3 weeks cumul	100.	208.048	{160., 257.}	111.254	0.324291	newly 5 weeks spec	36.	49.2698	{29., 72.}	47.0018	0.166867	newly 5 weeks cumul	184.	315.683	{251., 390.}	74.1546	0.240968	newly 58 weeks cumul	364.	534.873	{369., 746.}	49.3677	0.164597
49	newly 3 weeks spec	70.	68.4167	{27., 100.}	31.0238	0.204741	newly 3 weeks cumul	184.	204.817	{83., 295.}	38.7591	0.221225	newly 5 weeks spec	55.	58.95	{16., 99.}	49.6061	0.303283	newly 5 weeks cumul	319.	327.833	{153., 442.}	27.8788	0.186225	newly 62 weeks cumul	565.	572.883	{199., 824.}	34.4808	0.228997
50	newly 3 weeks spec	37.	41.82	{9., 71.}	52.9189	0.289786	newly 3 weeks cumul	82.	113.32	{26., 190.}	70.0488	0.294322	newly 5 weeks spec	52.	47.66	{9., 71.}	32.8077	0.240719	newly 5 weeks cumul	186.	206.36	{53., 337.}	45.1613	0.238925	newly 60 weeks cumul	449.	592.5	{373., 755.}	43.951	0.20425
